# SARS-CoV-2 Infects Human ACE2-Negative Endothelial Cells through an α_v_β_3_ Integrin-Mediated Endocytosis Even in the Presence of Vaccine-Elicited Neutralizing Antibodies

**DOI:** 10.3390/v14040705

**Published:** 2022-03-29

**Authors:** Antonella Bugatti, Federica Filippini, Marta Bardelli, Alberto Zani, Paola Chiodelli, Serena Messali, Arnaldo Caruso, Francesca Caccuri

**Affiliations:** 1 Section of Microbiology, Department of Molecular and Translational Medicine, University of Brescia, 25123 Brescia, Italy; antonella.bugatti@unibs.it (A.B.); f.filippini020@studenti.unibs.it (F.F.); m.bardelli@unibs.it (M.B.); a.zani033@unibs.it (A.Z.); s.messali@unibs.it (S.M.); arnaldo.caruso@unibs.it (A.C.); 2 Section of General Pathology, Department of Molecular and Translational Medicine, University of Brescia, 25123 Brescia, Italy; paola.chiodelli@unibs.it

**Keywords:** SARS-CoV-2 variants, endothelial cell dysfunction, RGD motif, α_v_β_3_ integrin, BNT162b2 vaccine

## Abstract

Integrins represent a gateway of entry for many viruses and the Arg-Gly-Asp (RGD) motif is the smallest sequence necessary for proteins to bind integrins. All Severe Acute Respiratory Syndrome Virus type 2 (SARS-CoV-2) lineages own an RGD motif (aa 403–405) in their receptor binding domain (RBD). We recently showed that SARS-CoV-2 gains access into primary human lung microvascular endothelial cells (HL-mECs) lacking Angiotensin-converting enzyme 2 (ACE2) expression through this conserved RGD motif. Following its entry, SARS-CoV-2 remodels cell phenotype and promotes angiogenesis in the absence of productive viral replication. Here, we highlight the α_v_β_3_ integrin as the main molecule responsible for SARS-CoV-2 infection of HL-mECs via a clathrin-dependent endocytosis. Indeed, pretreatment of virus with α_v_β_3_ integrin or pretreatment of cells with a monoclonal antibody against α_v_β_3_ integrin was found to inhibit SARS-CoV-2 entry into HL-mECs. Surprisingly, the anti-Spike antibodies evoked by vaccination were neither able to impair Spike/integrin interaction nor to prevent SARS-CoV-2 entry into HL-mECs. Our data highlight the RGD motif in the Spike protein as a functional constraint aimed to maintain the interaction of the viral envelope with integrins. At the same time, our evidences call for the need of intervention strategies aimed to neutralize the SARS-CoV-2 integrin-mediated infection of ACE2-negative cells in the vaccine era.

## 1. Introduction

Integrins are multifunctional, heterodimeric cell-surface adhesion molecules. They are receptors of extracellular ligands and transduce biochemical signals into the cell through downstream effector proteins. They are internalized and enter the endo/exocytic pathway before being recycled back to the plasma membrane [1]. The trafficking of these proteins is modulated by multiple context-dependent pathways, such as (i) clathrin-mediated endocytosis [2], (ii) caveolae-mediated endocytosis [3], and (iii) clathrin-caveolae-independent endocytosis [4].

Integrins play important roles in cell proliferation, migration, apoptosis, tissue repair, as well as in all processes critical to inflammation, infection, and angiogenesis. They represent a gateway of entry for many viruses that successfully infect host cells using integrin-mediated endocytic pathways [1,5]. Indeed, different viruses present the Arg-Gly-Asp (RGD) motif allowing their interaction and internalization through integrins [6,7,8]. The RGD motif is the smallest peptide sequence necessary for proteins to bind integrins. Although Angiotensin-converting enzyme 2 (ACE2) is the main receptor for Severe Acute Respiratory Syndrome Virus type 2 (SARS-CoV-2) entry into target cells, the viral Spike protein also owns a RGD motif (aa 403–405), which is peculiar for all SARS-CoV-2 lineages known to date and absent in all the other coronavirus Spike proteins [9]. It has been proposed that the RGD domain may increase the binding strength of SARS-CoV-2 Spike to ACE2 and improve virus entry into host cells [10]. On the other hand, it may play a role in sustaining SARS-CoV-2 entry into ACE2-negative cells.

We recently demonstrated a direct role played by SARS-CoV-2-infected primary human lung microvascular endothelial cells (HL-mECs) in supporting vascular dysfunction upon infection [11]. Indeed, we showed that SARS-CoV-2 gains access through the conserved RGD motif in HL-mECs lacking ACE2 expression [11]. Following its entry, SARS-CoV-2 remodels cell phenotype and promotes angiogenesis in the absence of productive viral replication [11]. In particular, the presence of SARS-CoV-2 proteins into HL-mECs were found to induce the release of a multiplicity of pro-inflammatory and pro-angiogenic molecules. Moreover, the SARS-CoV-2-conditioned microenvironment was able to induce uninfected HL-mEC to acquire a pro-angiogenic phenotype.

Here, we use HL-mECs for a deeper understanding of the role of integrins in SARS-CoV-2 entry into ACE2-negative cells. We highlight α_v_β_3_ as the main integrin molecule responsible for SARS-CoV-2 binding and entry into HL-mEC via a clathrin-dependent endocytosis. Inhibition of α_v_β_3_/Spike protein interaction was sufficient to inhibit the SARS-CoV-2-sustained endothelial cell (EC) dysfunction.

Surprisingly, anti-Spike antibodies evoked by vaccination were not able to affect integrins/Spike interaction and therefore, SARS-CoV-2 infection.

## 2. Materials and Methods

### 2.1. Cells

African green monkey kidney Vero E6 cell line (Istituto Zooprofilattico Sperimentale della Lombardia e dell’Emilia Romagna, Brescia, Italy) were cultured in Dulbecco’s Modified Eagle Medium (DMEM; Gibco, Thermo Fisher Scientific, Waltham, MA, USA) supplemented with 10% Fetal Bovine Serum (FBS; Gibco, Thermo Fisher Scientific). HL-mECs (Lonza Clonetics, Walkersville, MD, USA) were maintained in EGM-2 MV (Lonza, Basel, Switzerland) containing 10% FBS. A549 ACE2-positive (A549 ACE2^+^) cells, a kind gift from Dr. Stephen J Elledge (Harvard Medical School, Boston, MA, USA), were cultured in RPMI (Gibco, Thermo Fisher Scientific) supplemented with 10% FBS (Gibco, Thermo Fisher Scientific).

### 2.2. Viral Infection

Infections were carried out using the clinical SARS-CoV-2 isolate AP66 previously described [12,13] named B.1, or the SARS-CoV-2 variants (B.1.617.2 isolate hCoV-19/Italy/UNIBS-EC04-PV/2021, named B.1.617.2; B.1.1 isolate hCoV-19/Italy/UNIBS-MB61-P1V/2020 named B.1.1; B.1.1.7 isolate hCoV-19/Italy/UNIBS-DB00-P1V/2020 named B.1.1.7; B.1.351 isolate hCoV-19/Italy/UNIBS-MG73-P1V/2021 named B.1.351; B.1.525 isolate hCoV-19/Italy/UNIBS-KA93-P1V/2021 named B.1.525 and B.1.621 isolate hCoV-19/Italy/LOM-UNIBS-DT04-P1V/2021 named B.1.621). The viruses were propagated in Vero E6 cells and the viral titer was determined by a standard plaque assay. All the experiments were performed with a single viral inoculum. Mock-infected cell cultures were obtained from uninfected cells, processed exactly as the SARS-CoV-2-infected ones. All the infection experiments were carried-out in a biosafety level-3 (BLS-3) laboratory at a Multiplicity of Infection (MOI) of 1. When indicated, HL-mECs were pretreated for 1 h at 37 °C with 30 µg/mL of GRGDSPK (RGD) or GRADSPK (RAD) peptides (Merck, Darmstadt, Germany) before SARS-CoV-2 infection. When reported, HL-mECs or A549 ACE2^+^ cells were pretreated for 1 h at 37 °C with or without 100 µg/mL of mAb against α_v_β_3_ (Bio-techne, Minneapolis, MN, USA) before SARS-CoV-2 infection. When indicated, the virus was pretreated for 1 h at 37 °C with 5 µg/mL of soluble α_v_β_3_ integrin (Bio-techne) and then used to infect HL-mECs or A549 ACE2^+^ cells.

### 2.3. Authentic Virus Neutralization Assay

Authentic virus neutralization assay was performed as previously described, with minor modification [14]. HL-mECs or A549 ACE2^+^ cells were seeded in 48-well plates (5 × 10^4^ and 5 × 10^5^ cells per well, respectively) in complete medium. Prior to infection, SARS-CoV-2 belonging to B.1.617.2 lineage was preincubated or not for 1 h with sera (1:100) collected from volunteers who received the third dose of the BNT162b2 vaccine. Then, the virus–serum mixture was used to infect HL-mECs or A549 ACE2^+^ cells. Twenty-four h post infection (p.i.), the presence of SARS-CoV-2 mRNA at an intracellular level was evaluated by qRT-PCR.

### 2.4. Viral RNA Extraction and qRT-PCR

RNA was extracted from clarified cell culture supernatants and infected cells using QIAamp Viral RNA^®^ Mini Kit (Qiagen, Hilden, Germany) and RNeasy Plus mini kit (Qiagen), respectively, according to the manufacturer’s instructions. The qRT-PCR was carried out following previously described procedures [13]. Briefly, reverse transcription and amplification of the spike (S) gene were performed using the one-step QuantiFast Sybr Green RT-PCR mix (Qiagen) as follows: 50 °C for 10 min, 95 °C for 5 min; 95 °C for 10 sec, 60 °C for 30 sec (40 cycles) (primers: RBD-qF1: 5′-CAA TGG TTT AAC AGG CAC AGG-3′ and RBD-qR1: 5′-CTC AAG TGT CTG TGG ATC ACG-3). A standard curve was obtained by cloning the receptor-binding domain of the S gene (primers: RBD-F: 5′-GCT GGA TCC CCT AAT ATT ACA AAC TTG TGC C-3′; RBD-R: 5′-TGC CTC GAG CTC AAG TGT CTG TGG ATCAC-3′) into pGEM T-easy vector (Promega, Madison, WI, USA). A standard curve was generated by the determination of copy numbers derived from serial dilutions (10^3^–10^9^ copies) of the plasmid. Each quantification was run in triplicates.

### 2.5. Immunofluorescence Assay

For the evaluation of α_v_β_3_ and α_5_β_1_ expression, HL-mECs or A549 ACE2^+^ cells (5 × 10^4^ cells per experimental condition) were fixed with 4% paraformaldehyde (PFA) in Phosphate Buffered Saline (PBS) for 10 min, permeabilized or not with 0.1% Triton X-100 in PBS, and saturated with 3% Bovine Serum Albumin (BSA) in PBS. For staining, cells were incubated 1 h with mAb against α_v_β_3_ integrin (1 µg/mL; Bio-techne) and α_5_β_1_ integrin (1 µg/mL; Thermo Fisher Scientific) followed by Alexa Fluor 488-conjugated anti-mouse IgG or Alexa Fluor 594-conjugated anti-rabbit IgG (Thermo Fisher Scientific, Waltham, MA, USA). Nuclei were counterstained with 4′,6-diamidino,2-phenylindole (DAPI, Merck, Darmstadt, Germany). For the evaluation of Spike-α_v_β_3_ localization: HL-mECs cells were seeded (5 × 10^4^ cells per well) on collagen-coated 8-well chamber slides (Thermo Fisher Scientific) in complete medium. After 24 h, cells were incubated at 4 °C for 1 h in appropriate media with 15% FBS containing recombinant Spike of SARS-CoV-2 (100 ng/mL; Bio-techne) (pulse phase). After incubation, the cells were washed and then incubated at 37 °C (or at 4 °C for cellular surface Spike-α_v_β_3_ localization) in appropriate media with 15% FBS in the absence of Spike protein (chase phase). After 30 min, the cells were washed, fixed with 4% PFA in PBS for 10 min, permeabilized or not with 0.1% Triton X-100 in PBS, and saturated with 3% BSA in PBS. For staining, the cells were incubated for 1 h with a human serum (collected from a volunteer who received the third dose of the BNT162b2 vaccine; 1:1000 dilution) containing IgG to the SARS-CoV-2 Spike protein followed by Alexa Fluor 488-conjugated anti-human IgG (Thermo Fisher Scientific). Nuclei were counterstained with DAPI (Merck). Cells were photographed under a Zeiss Axiovert 200 M epifluorescence microscope equipped with a Plan-Apochromat 40Xor 63X/1.4 NA oil objective (Zeiss Axiovert 200M system).

In some experiments, HL-mECs were seeded (5 × 10^4^ cells per well) on collagen-coated 8-well chamber slides (Thermo Fisher Scientific) and infected with SARS-CoV-2, belonging to the B1.617.2 lineage, as previously described. After infection, cells were fixed with 4% paraformaldehyde in PBS for 10 min, permeabilized with 0.1% Triton X-100 in PBS, and saturated with 3% BSA, 0.1% Tween 20 in PBS. For staining, cells were incubated for 1 h with a human serum (collected from a volunteer who received the third dose of the BNT162b2 vaccine; 1:1000 dilution) containing IgG to SARS-CoV-2, followed by Alexa Fluor 488-conjugated anti-human, IgG (Thermo Fisher Scientific). Nuclei were counterstained with DAPI. Fluorescence was recorded with a Nikon DXM 1200 digital camera system (Nikon, Minato, Tokyo, Japan) coupled to the Eclipse E1000 fluorescence microscope (Nikon) and the ACT-1 control software (Nikon). Endocytosis was inhibited by incubation with Monensin (2 µM, Merck) or Brefeldin A (3 µg/mL; Merck). Cathepsin-mediated proteolysis was prevented by the addition of 20 µM Cathepsin L inhibitor III (Calbiochem, Merck).

### 2.6. Flow Cytometry

HL-mECs or A549 ACE2^+^ cells (5 × 10^5^ cells per experimental condition) were fixed with 4% PFA in PBS. For staining, cells were incubated 1 h with mAb directed against α_v_β_3_ integrin (5 µg/mL; Bio-techne) or α_5_β_1_ integrin (5 µg/mL; Thermo Fisher Scientific) followed by Alexa Fluor 488-conjugated anti-mouse IgG or Alexa Fluor 488-conjugated anti-rabbit IgG (Thermo Fisher Scientific). Fluorescence was analyzed by flow cytometry, and the results were expressed as the percentage of positive cells, compared to isotype-stained samples. Data were analyzed by FlowJo software 10.0 version (Tree Star Inc., Ashland, OR, USA).

### 2.7. Tube Formation Assay

Tube formation was performed as previously described [11]. Briefly, 150 µL of Cultrex Reduced Growth Factor Basement Membrane Extract (RGF BME) (Trevigen, Inc., Gaithersburg, MD, USA) were transferred to prechilled 48-well culture plates. Plates were incubated for 1 h at 37 °C. Cells were resuspended in the culture medium containing 10% FBS, seeded 4.5 × 10^4^ per well, and analyzed for tube formation at 12 h after cell seeding by examination with a Leica DM IRB microscope (Leica, Wetzlar, Germany). Vascular Endothelial Growth Factor-A (VEGF-A) (30 ng/mL, Bio-techne) was used as a positive control. The center of each well was digitally photographed with a Hitachi KP-D50 camera (Hitachi, Chiyoda, Tokyo, Japan) and capillary-like structures were quantified by analyzing the number of tubes per well formed by ECs.

### 2.8. Spheroids Assay

Spheroids were generated as previously described [11]. Briefly, HL-mECs (2 × 10^5^ cells/mL) infected with SARS-CoV-2, belonging to the B.1.617.2 lineage, were mixed with 5 mg/mL of methylcellulose (Sigma-Aldrich, Inc., St. Louis, MO, USA) in EGM-2 MV medium containing 10% FBS, taking the final volume to 10 mL. The cells (100 µL/well) were then added to 96-well plates (Greiner Bio-one, Kremsmünster, Austria) and incubated at 37 °C, 5% CO_2_ for 24 h. Separately, the collagen I gel solution (Rat tail, Corning Incorporated, Corning, NY, USA) was maintained on ice and neutralized by adding NaOH 0.1 M and PBS 10X to a final pH of 7.4. Then, the 24-well plates were coated with neutralized collagen (200 µL/well) and incubated in a humidified 5% CO_2_ incubator for 1 h at 37 °C. The spheroids from 96-well plates were collected in eppendorf tubes and centrifuged at 4000 rpm for 10 sec. When a clear pellet was distinguished, the supernatant was removed, and the pellet was kept in a volume of about 100 µL collagen I-neutralized solution. Each collagen-spheroid mixture was rapidly added to the precoated 24-well plates (100 µL/well) and incubated for 1 h. After 1 h, 500 µL of EGM-2 MV containing 10% FBS, was added to the wells to cover the surface completely and plates were further incubated for 24 h. Sprouting occurred from the spheroid core, photographed with a Hitachi KP-D50 camera, and the number of sprouts was counted in the spheroids of similar sizes from three different wells of the plate.

### 2.9. Microarray Analysis

Supernatants from SARS-CoV-2, belonging to the B.1.617.2 lineage, infected HL-mECs were collected at 3 days p.i., clarified and analyzed for the expression of 55 different angiogenesis-related proteins by Human Angiogenesis Array Kit (Proteome Profiler, R&D systems, Minneapolis, MN, USA) according to the manufacturer’s instructions.

### 2.10. Biacore

Surface Plasmon Resonance (SPR) measurements were conducted on a Biacore X100 (Cytiva, Washington DC, USA) at 25 °C. SPR was used to characterize the binding of α_v_β_3_ integrin to the RBD of Spike protein on a sensor chip. The entire RBD (223 amino acids; 20 µg/mL, Genaxxon Bioscience, Ulm, Germany) was immobilized onto a CM5 sensor chip allowing the immobilization of 224 resonance units (RU), equal to 9 fmol/mm^2^ of RBD. A sensor chip alone was used to evaluate nonspecific binding and for blank subtraction [15]. Increasing concentrations of integrin α_v_β_3_ (Bio-techne) in 10 mM Hepes pH 7.4 containing 0.15 mM NaCl, 50 µM EDTA, 0.005% Surfactant P20, 1 mM CaCl_2_, 1 mM MgCl_2_, and 1 mM MnCl_2_ (running buffer) were injected over the RBD or blank surfaces for 2 min and then washed until dissociation. After each run, the sensor chip was regenerated by injection of 2 M NaCl. Kinetic parameters were calculated from the sensorgram overlays by using the nonlinear fitting single-site model software package BIAevaluation (version 3.2 [Cytiva]). Only sensorgrams whose fitting gave χ^2^ values close to 10 were used [16]. Saturation curve was obtained by using the values of RU bound at equilibrium from injection of increasing concentrations of free α_v_β_3_ onto sensor chip immobilized RBD.

### 2.11. Statistical Analysis

Data were analyzed for statistical significance using the one-way ANOVA. Bonferroni’s post-test was used to compare data. Differences were considered significant when *p* < 0.05. Statistical tests were performed using Prism 8 software (GraphPad Software, La Jolla, CA, USA).

## 3. Results

### 3.1. Integrins Expression on HL-mECs

**Figure 1:**
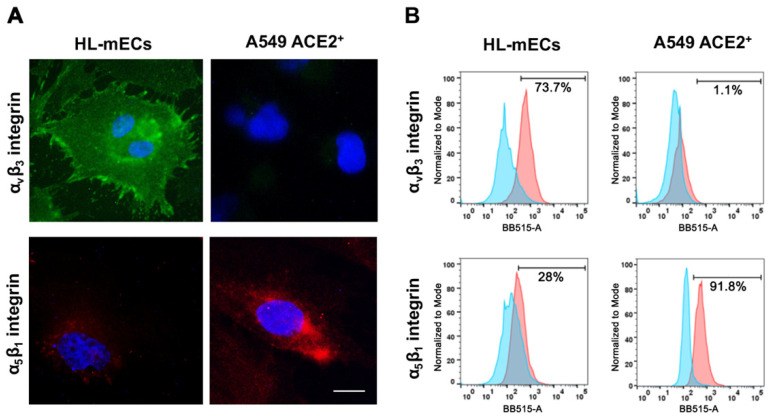
Surface expression of α_v_β_3_ and α_5_β_1_ integrins on HL-mECs and A549 ACE2^+^ cells. (**A**) Immunofluorescence visualization of α_v_β_3_ and α_5_β_1_ integrins on HL-mECs and A549 ACE2^+^ cells. Cells were fixed with 4% PFA and incubated with a monoclonal antibody against α_v_β_3_ or α_5_β_1_ integrins, followed by Alexa Fluor 488-conjugated anti-mouse IgG (green) or Alexa Fluor 594-conjugated anti-rabbit IgG (red). Nuclei were counterstained with DAPI (scale bar, 20 µm). (**B**) Flow cytometric analysis of α_v_β_3_ and α_5_β_1_ expression on HL-mECs and A549 ACE2^+^ cells. Cells were fixed with 4% PFA and incubated with a monoclonal antibody against α_v_β_3_ or α_5_β_1_ integrins and followed by Alexa Fluor 488-conjugated anti-human mouse IgG or Alexa Fluor 488-conjugated anti-rabbit IgG. Histograms show the rightward shift for the BB515-A + signal (red) compared to isotype control (light blue).

Many viruses utilize distinctive receptors to enter different target cells [5]. Integrins often represent entry receptors for a plethora of nonenveloped and enveloped viruses, such as Kaposi’s sarcoma-associated herpesvirus (KSHV), herpes simplex virus-2 (HSV-2), adenovirus and papillomavirus [5]. Nader and colleagues [17] showed that binding of SARS-CoV-2 Spike to α_v_β_3_ integrin was able to induce human aortic EC dysregulation. Other investigators pointed on the role of α_5_β_1_ in facilitating SARS-CoV-2 binding and entry in ACE2^+^ human cardiac myocytes [18]. More recently, we highlighted the role of integrins in SARS-CoV-2 entry into ACE2-negative HL-mECs [11]. To better define the mechanisms leading to SARS-CoV-2 entry into cells lacking ACE2 expression, at first we examined the basal level expression of both α_v_β_3_ and α_5_β_1_ integrins on the surface of HL-mECs as compared to A549 ACE2^+^ cells, used as control for SARS-CoV-2 infection in all our experimental sets. Immunofluorescence, carried out on fixed and not permeabilized cells using mAbs against α_v_β_3_ and α_5_β_1_, as specific reagents, revealed that both integrins are expressed on the surface of HL-mECs, even though to a different extent (Figure 1A). In particular, flow cytometric analysis showed that the expression of α_v_β_3_ in HL-mECs was significantly higher than that of α_5_β_1_ (Figure 1B). Differently, α_5_β_1_ was strongly expressed on the surface of A549 ACE2^+^ cells, whereas α_v_β_3_ was only barely detected (Figure 1B). These data suggest α_v_β_3_ integrin as the major player for SARS-CoV-2 entry into HL-mECs.

### 3.2. SARS-CoV-2 Variants Do Not Productively Infect HL-mECs

**Figure 2:**
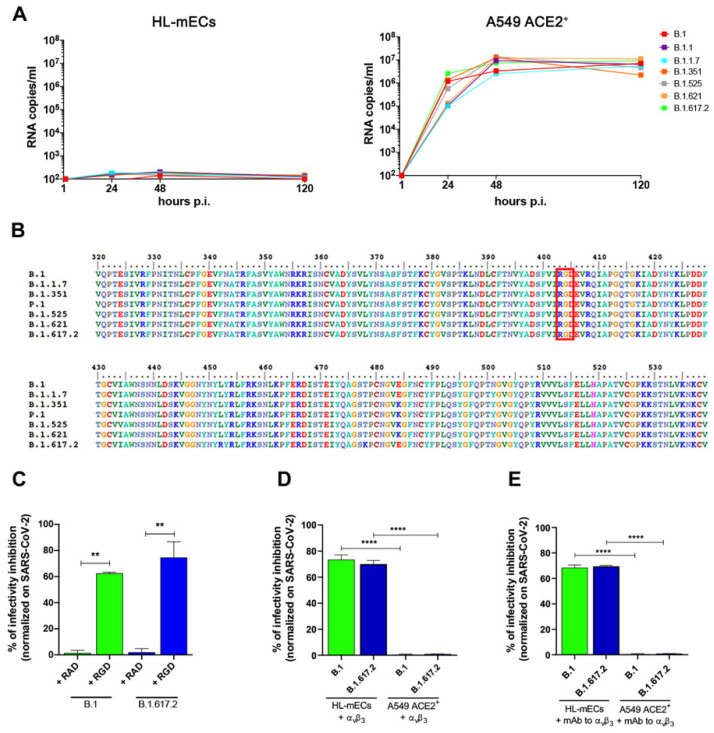
The RGD motif mediates SARS-CoV-2 variants entry into ACE2-negative cells. (**A**) HL-mECs (left panel) and A549-ACE2^+^ cells (right panel) were infected with SARS-CoV-2 belonging to B.1, B.1.1, B.1.1.7, B.1.351, B.1.525, B.1.621, and B.1.617.2 lineages at a MOI of 1. The graphs show SARS-CoV-2 genome quantitation in cell supernatants collected at 1, 24, 48, and 120 h p.i. by qRT-PCR. Data are representative of two independent experiments with similar results. (**B**) Schematic representation of the RBD of SARS-CoV-2 Spike protein. The RBD sequences of the different SARS-CoV-2 lineages were aligned. Numbers refer to the Spike protein sequence. The RGD motif is highlighted in red. (**C**) HL-mECs were pretreated with 30 µg/mL of RAD or RGD peptides before SARS-CoV-2 B.1 or B.1.617.2 infection. Twenty-four h p.i., the presence of SARS-CoV-2 mRNA at an intracellular level was evaluated by qRT-PCR. The percentage of infectivity inhibition was calculated by comparing SARS-CoV-2 treated cells and SARS-CoV-2 untreated cells. Data are representative of two independent experiments with similar results. Statistical analysis was performed by one-way ANOVA and Bonferroni’s post-test was used to compare data (** *p* < 0.01) by relating RAD- and RGD-treated cells. (**D**) Prior to infection, SARS-CoV-2 B.1 or B.1.617.2 were preincubated or not for 1 h, with α_v_β_3_ integrin (5 µg/mL). Then, the viruses were used to infect HL-mECs or A549 ACE2^+^ cells for 1 h at a MOI of 1. Twenty-four h p.i., the presence of SARS-CoV-2 mRNA at an intracellular level was evaluated by qRT-PCR. The percentage of infectivity inhibition was calculated by comparing cells infected with α_v_β_3_-treated SARS-CoV-2 and cells infected with untreated SARS-CoV-2. Data are representative of two independent experiments with similar results. Statistical analysis was performed by one-way ANOVA and Bonferroni’s post-test was used to compare data (**** *p* < 0.0001) by relating HL-mECs and A549 ACE2^+^ cells. (**E**) HL-mECs or A549 ACE2^+^ cells were pretreated or not with mAb against α_v_β_3_ integrin (100 µg/mL) for 1 h at 37 °C. Then, mAb pretreated cells were infected, for 1 h, with SARS-CoV-2 B.1 or B.1.617.2 at a MOI of 1. Twenty-four h p.i., the presence of SARS-CoV-2 mRNA at an intracellular level was evaluated by qRT-PCR. The percentage of infectivity inhibition was calculated by comparing SARS-CoV-2-treated cells and SARS-CoV-2-untreated cells. Data are representative of two independent experiments with similar results. Statistical analysis was performed by one-way ANOVA and Bonferroni’s post-test was used to compare data (**** *p* < 0.0001) by relating HL-mECs and A549 ACE2^+^ cells.

Many emerging viruses share the ability to infect ECs, thus contributing to dissemination of the infection to many tissues and organs [19]. The most common feature observed in severe SARS-CoV-2 infection relates to EC dysfunction [20,21]. Our previous data showed that SARS-CoV-2 belonging to the B.1 lineage induces an abortive infection of HL-mECs, which results in changes in cell phenotype fostering angiogenic functions [11]. Since autumn 2020, a consistent number of SARS-CoV-2 variants have emerged and spread globally. In order to understand if any of these variants could productively infect HL-mECs, we used SARS-CoV-2 belonging to B.1.1, B.1.1.7, B.1.351, B.1.525, B.1.621, and B.1.617.2 lineages along with the B.1 lineage to infected HL-mECs, at high (1) MOI. As shown in Figure 2A (left panel), SARS-CoV-2 RNA levels released by infected HL-mECs did not increase over time, demonstrating that none of the tested SARS-CoV-2 variants were able to induce active virus replication in HL-mECs. As expected, A549 ACE2^+^ cells infected in parallel to HL-mECs with the same SARS-CoV-2 variants inoculum released an abundant amount of viral RNAs (Figure 2A, right panel). These data demonstrate that HL-mECs are not productively infected by SARS-CoV-2 variants.

### 3.3. α_v_β_3_ Integrin Mediates SARS-CoV-2 Entry into HL-mECs

The RGD motif is present in the Spike protein of all SARS-CoV-2 lineages known to date (Figure 2B), but absent in all other coronavirus Spike proteins [9,22]. Previously, we demonstrated the involvement of integrins in SARS-CoV-2 entry into HL-mECs by using the peptide RGD as an inhibitor of integrin–ligand interactions [11]. Thus, we wondered whether such motif could also play a role in SARS-CoV-2 variants entry into HL-mECs. To test this hypothesis, at first we used the RGD peptide to treat cells prior to infection with the SARS-CoV-2 B.1.617.2 lineage, the most diffuse and aggressive variant up to date. In parallel, we carried out infection with a viral isolate belonging to the SARS-CoV-2 B.1 lineage. As shown in Figure 2C, a significant inhibition of SARS-CoV-2 B.1 and B.1.617.2 entry (62.6% and 74.6%, respectively) was observed in the RGD-treated HL-mECs, as compared to the untreated cells. As expected, SARS-CoV-2 entry was not significantly affected by pretreatment with the RAD peptide, thus highlighting the role played by integrins in mediating SARS-CoV-2 entry into HL-mECs.

The ability of SARS-CoV-2 to infect the endothelium, together with the evidence that this infection induces endothelial dysfunction, underscores the need for a greater understanding of the mechanisms leading virus entry into ECs. To gain deeper insight into the major players involved in SARS-CoV-2 Spike/integrins interaction, we performed SARS-CoV-2 infection upon inhibition of α_v_β_3_ integrin.

To this end, we incubated SARS-CoV-2 B.1 and B.1.617.2 with α_v_β_3_ integrin for 1 h at 37 °C; the viruses were then used to infect either HL-mECs or A549 ACE2^+^ cells. As shown in Figure 2D, entry of both viruses into HL-mECs was strongly inhibited by integrin pretreatment (73.5% and 70%, respectively). As expected, SARS-CoV-2 entry into A549 ACE2^+^ cells was not affected by α_v_β_3_ pretreatment. To further confirm these data, we performed a similar experiment by preincubating cells with mAb against α_v_β_3_ for 1 h at 37 °C. As shown in Figure 2E, binding of mAb to α_v_β_3_ expressed on HL-mECs surface resulted in a strong inhibition of SARS-CoV-2 B.1 and B.1.617.2 entry (73% and 69.5%, respectively). On the contrary, SARS-CoV-2 B.1 and B.1.617.2 entry was not affected when A549 ACE2^+^ cells were pretreated with mAb against α_v_β_3,_ which is not expressed on A549 ACE2^+^ cells (Figure 1). Taken together, these data demonstrate that the RGD motif is a constraint well preserved among SARS-CoV-2 variants and highlight the main involvement of α_v_β_3_ in SARS-CoV-2 entry into HL-mECs.

### 3.4. α_v_β_3_ Integrin Is Involved in SARS-CoV-2-Triggered Angiogenic Functions

**Figure 3:**
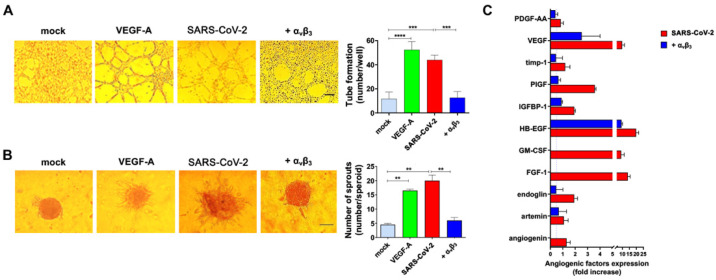
α_v_β_3_ counteracts SARS-CoV-2 proangiogenic effects. HL-mECs were mock-infected (mock) or infected with SARS-CoV-2 belonging to the B.1.617.2 lineage (SARS-CoV-2) or with α_v_β_3_-treated B.1.617.2 (+α_v_β_3_) at MOI 1, for 1 h at 37 °C and then washed and cultured until day 3 p.i. (**A**) Mock, SARS-CoV-2, and + α_v_β_3_ HL-mECs were seeded on reduced growth factor Matrigel-coated wells and then cultured for 12 h at 37 °C. Pictures are representative of one out of three independent experiments with similar results (scale bar, 200 μm). VEGF-A was used as a positive control. Values reported in the graph are the mean ± SD of one representative experiment out of three independent experiments with similar results performed in triplicate. Statistical analysis was performed by one-way ANOVA and Bonferroni’s post-test was used to compare data (*** *p* < 0.001; **** *p* < 0.0001). (**B**) Sprouting of spheroids generated with Mock, SARS-CoV-2, or +α_v_β_3_ HL-mECs. Pictures are representative of one out of three independent experiments with similar results (scale bar, 10 μm). VEGF-A was used as a positive control. Values reported in the graph are the mean ± SD of one representative experiment out of three independent experiments with similar results performed in triplicate. Statistical analysis was performed by one-way ANOVA and Bonferroni’s post-test was used to compare data (** *p* < 0.01). (**C**) Human proteome array for angiogenic molecules. The results are expressed as mean values ± SD of duplicates given as fold increase, as compared to mock-infected cells. Data are representative of one out of three independent experiments with similar results.

Integrins not only represent receptors expressed on the surface of cells which can be utilized by viruses for attachment and entry into cells [5], but are also critical for the pathology of virus-induced conditions. For instance, KSHV can infect cells through an RGD motif expressed on its gB glycoprotein [23], leading to the activation of several signaling pathways [24]. Previously, we demonstrated that SARS-CoV-2 infection induces a phenotype remodeling of HL-mECs, leading to an angiogenic microenvironment [11]. At least six integrins have been identified as contributor of angiogenic processes [25,26], among which α_v_β_3_ has been highlighted as a positive regulator of the angiogenic switch [27,28]. To gain deeper understanding of the role played by α_v_β_3_ integrin in promoting SARS-CoV-2-mediated angiogenic functions, we carried out two different assays. We first examined the capacity of HL-mECs to form tube-like structures upon infection with α_v_β_3_ integrin-pretreated or not virus inocula. To this end, cells were collected 3 days p.i. and seeded on 48-well plates containing polymerized plugs of growth factor-reduced BME. As shown in Figure 3A, SARS-CoV-2 B.1.617.2-infected HL-mECs (SARS-CoV-2) formed a consistent network of tube-like structures, almost superimposable to that observed after stimulation with a potent proangiogenic factor such as VEGF-A. At the same time, HL-mECs formed mainly a cellular monolayer when infected with α_v_β_3_-pretreated SARS-CoV-2 virus (+α_v_β_3_), showing the same phenotype of mock-infected cells (mock). To further confirm this data, we took advantage of the spheroids assay, a three-dimensional (3D) cell model that mimics in vivo sprouting angiogenesis [29]. Three days p.i., HL-mECs infected with SARS-CoV-2 pretreated or not with α_v_β_3_ integrin were collected to generate spheroids [11,30,31]. After 24 h of observation, mock-infected spheroids (mock) did not develop sprouts, while in SARS-CoV-2 B.1.617.2-infected spheroids (SARS-CoV-2), a consistent outgrowth of sprouts was observed (Figure 3B). The effect of SARS-CoV-2 infection on sprouting angiogenesis was found to be superimposable to that observed using VEGF-A as a positive control of the assay. Interestingly, spheroids generated by using cells infected with α_v_β_3_-pretreated SARS-CoV-2 (+α_v_β_3_) showed a dramatic reduction in vessels’ outgrowth.

Pine and colleagues [32] correlated circulating vascular markers with disease severity. For instance, angiogenesis markers were found to increase in hospitalized patients, and markers of endotheliopathy were higher in those patients who died from COVID-19. Our recent data highlighted the release of a plethora of angiogenic molecules from SARS-CoV-2-infected HL-mECs [11]. To understand if the inhibition of SARS-CoV-2-induced angiogenic activity operated by pretreatment of virus with α_v_β_3_ integrin could impair the release of angiogenic molecules, we performed a secretome analysis at day 3 p.i. by using a human angiogenic array. As expected, SARS-CoV-2 triggered the secretion of a multiplicity of proangiogenic molecules (Figure 3C, red bars), which was strongly inhibited by pretreatment of SARS-CoV-2 with α_v_β_3_ integrin (Figure 3C, blue bars). Collectively, these data show that inhibition of RGD/α_v_β_3_ interaction is *per se* capable of reverting SARS-CoV-2-induced EC dysfunction.

### 3.5. Neutralizing Antibodies Evoked by Vaccination Do Not Interfere with SARS-CoV-2 Entry into HL-mECs

**Figure 4:**
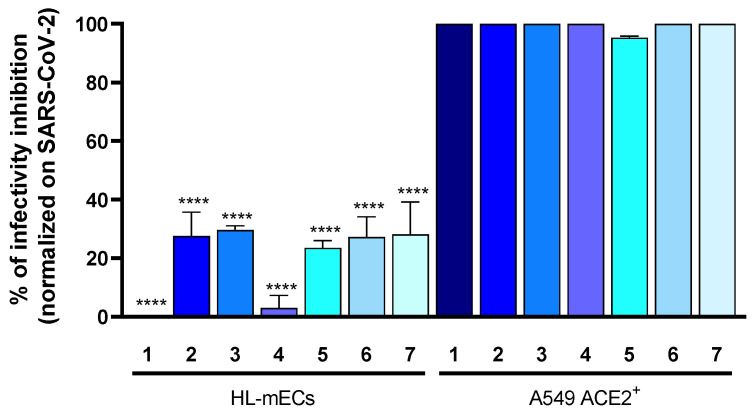
Vaccine elicited antibodies do not inhibit integrin-mediated SARS-CoV-2 entry into HL-mECs. Prior to infection, SARS-CoV-2 belonging to B.1.617.2 lineage was preincubated or not for 1 h, with sera (1:100) collected from volunteers after completing the BNT162b2 vaccine schedule (from 1 to 7). Then, the mixture virus-serum was used to infect HL-mECs or A549 ACE2^+^ cells. Twenty-four h p.i., the presence of SARS-CoV-2 mRNA at intracellular level was evaluated by qRT-PCR. The percentage of infectivity inhibition was calculated by comparing cells infected with serum-treated SARS-CoV-2 and cells infected with untreated SARS-CoV-2. Data are representative of two independent experiments with similar results. Statistical analysis was performed by one-way ANOVA and Bonferroni’s post-test was used to compare data (**** *p* < 0.0001) by relating HL-mECs and A549 ACE2^+^ cells.

The target of neutralizing antibodies elicited by either infection or vaccination against SARS-CoV-2 is represented by the RBD of the Spike protein, which contains three main antigenic sites [33]. The RBD-neutralizing antibodies mostly demonstrated an extraordinary potency to block ACE2 engagement by direct competition [34,35]. The epitope profiles of BNT162b2 vaccine-elicited antibodies showed that the epitope endowing the RGD motif is reactive at the highest level in the majority of the serum samples [36]. However, antibodies binding to this epitope demonstrated a negligible effect in neutralizing the live virus [36]. Thus, we wondered whether the antibody response induced by vaccination could inhibit SARS-CoV-2 entry into HL-mECs. To address this question, we tested seven human sera collected from volunteers who received the third dose of the BNT162b2 vaccine. SARS-CoV-2 was preincubated with each serum and the virus-antibody mixture was then used to infect HL-mECs or A549 ACE2^+^ cells. As shown in Figure 4, SARS-CoV-2 entry into HL-mECs was not significantly affected by any of the tested sera, whereas all the sera completely abolished viral entry into A549 ACE2^+^ cells, used as a control. According to Nitahara and colleagues [36], our data indicate that neutralizing antibodies evoked by vaccination against Spike directly compete with SARS-CoV-2 in order to block ACE2 binding, whereas they do not interfere with the integrins recognition site.

### 3.6. SARS-CoV-2 Spike-α_v_β_3_ Interaction Allows Virus Binding and Internalization

**Figure 5:**
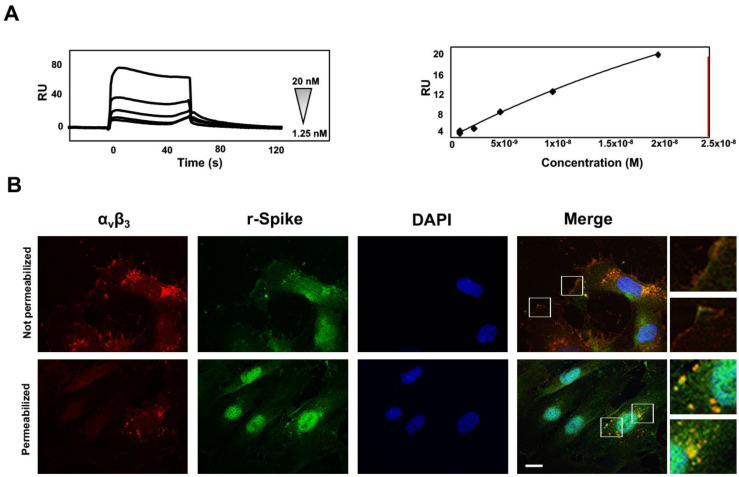
Recombinant SARS-CoV-2 Spike protein binds to α_v_β_3_ integrin and then is internalized. (**A**) Left panel, sensogram overlay showing the binding of increasing amounts of α_v_β_3_ integrin (from 1.25 to 20 nM) to immobilized entire RBD of SARS-CoV-2 Spike protein. The response, in resonance units (RU), was recorded as a function of time. Right panel, saturation curve obtained by using the values of RU bound at equilibrium from injection of increasing concentrations of free α_v_β_3_ onto sensor chip immobilized entire RBD. (**B**) HL-mECs were incubated at 4 °C in RPMI (15% FBS) containing 100 ng/mL of recombinant SARS-CoV-2 Spike protein. After 1 h of incubation, cells were washed and then incubated at 37 °C in RPMI (15% FBS) in the absence of Spike protein. After 30 min, cells were washed, fixed with 4% PFA, permeabilized (lower panel) or not (upper panel) with PBS 0.1% Triton X-100, saturated with 0.1% BSA, 0.1% Tween 20 in PBS and probed with a human serum containing IgG to SARS-CoV-2 and with a mouse monoclonal antibody against α_v_β_3_ integrin followed by Alexa Fluor 488-conjugated anti-human mouse IgG and by 594 conjugated anti-mouse goat IgG1. Magnified views on the upper panels show α_v_β_3_ expression (red) and the recombinant Spike protein (green) on cellular surface (not permeabilized). Magnified views on the lower panels show α_v_β_3_ and the recombinant Spike proteins internalized in HL-mECs (permeabilized). Boxes indicate the regions in the merged pictures that are magnified three-fold to show the localization of Spike protein and α_v_β_3_ integrin (scale bar, 50 µm).

Differently from other cell-surface receptors which are internalized and degraded, integrins are continuously trafficked in cells [1]. Integrins can be endocytosed through different mechanisms and the clathrin-mediated endocytosis is the most studied integrin internalization path [37]. Some viruses, such as adenovirus, interact with the α_v_β_3_ integrin, and then are internalized [7] mostly via clathrin-coated vesicles [8]. To elucidate the mechanisms leading to SARS-CoV-2 entry into HL-mECs, we first scrutinized the binding of Spike to the α_v_β_3_ integrin. For this purpose, we performed SPR analyses by using recombinant proteins as specific reagents. In particular, the entire RBD domain of the Spike protein was immobilized on a BIAcore CM5 sensor chip, while a sensor chip alone was used to evaluate nonspecific binding and for blank subtraction. The RBD/α_v_β_3_ interaction was found to be dose-dependent with a high kinetic affinity value, equal to 6.3 nM (Figure 5A, left panel). As shown in Figure 5A (right panel), measurement of steady-state binding levels showed an affinity value equal to 59.3 nM.

Immunofluorescence assay was carried out to evaluate internalization and intracellular localization of the Spike protein in HL-mECs. In particular, cells were treated for 1 h with a recombinant Spike protein and then fixed and permeabilized or not before staining with a human serum containing IgG to SARS-CoV-2 Spike. To avoid internalization, not-permeabilized cells were maintained on ice during the staining step. Localization of the α_v_β_3_ and Spike was not observed inside not-permeabilized HL-mECs (Figure 5B, upper panels), whereas it occurred in permeabilized HL-mECs (Figure 5B, lower panels).

### 3.7. α_v_β_3_-Mediated SARS-CoV-2 Entry Is Regulated by an Endocytic Pathway

**Figure 6:**
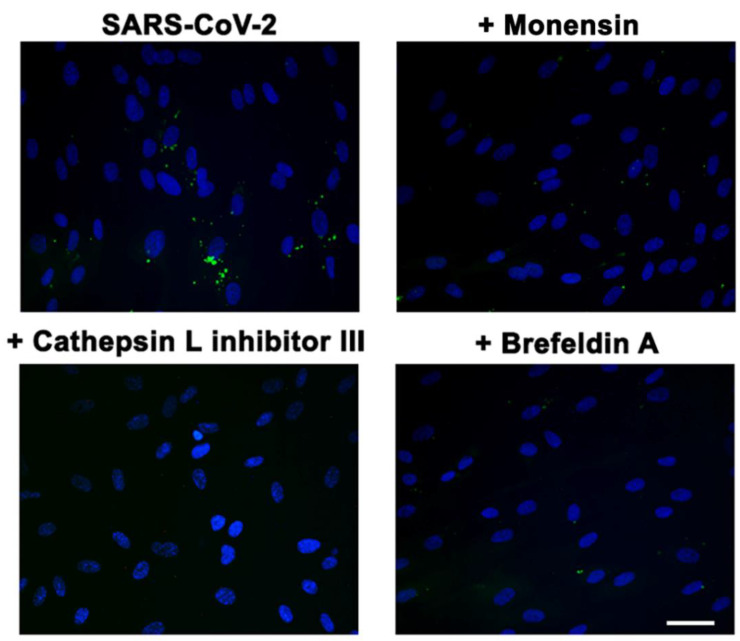
SARS-CoV-2 enters into HL-mECs through endocytosis. HL-mECs were pretreated for 1 h at 37 °C with Cathepsin L inhibitor III (20 µM), Monensin (2 µM), and Brefeldin A (3 µg/mL). After treatment, cells were infected with SARS-CoV-2 belonging to B.1.617.2 lineage. Twenty-four h p.i., cells were fixed with 4% PFA in PBS and permeabilized with 0.1% Triton X-100 in PBS, and saturated with 0.1% BSA, 0.1% Tween 20 in PBS. For staining, cells were incubated for 1 h with a human serum containing IgG to SARS-CoV-2 (1:1000 dilution) followed by Alexa Fluor 488-conjugated anti-human mouse IgG. Nuclei were counterstained with DAPI (scale bar, 20 µm).

It has been demonstrated that cleavage of Spike operated by both furin and TMPRSS2 proteases is a prerequisite for efficient SARS-CoV-2 infection of lung epithelial cells [38,39,40]. These two proteases work in concert, but it has been demonstrated that SARS-CoV-2 may gain access into target cells also in the absence of furin and TMPRSS2 following an endocytic pathway, which can be activated by lysosomal cathepsin L [41]. It is well known that many viruses, after binding to integrins, gain access into target cells through an endocytic pathway [5]. Thus, we hypothesize that the internalization of SARS-CoV-2 into ACE2-negative cells could be ascribed to an endocytic mechanism. To this end, we carried out SARS-CoV-2 infection on HL-mECs after their pretreatment with different inhibitors of the endocytic pathways. We used Cathepsin L inhibitor III that inhibits the endosomal cysteine proteases Cathepsin L [42], and two inhibitors of the clathrin-mediated endocytosis, namely Monensin and Brefeldin A [43,44]. As shown in Figure 6, HL-mECs pretreatment with Cathepsin L inhibitor III, as well as with Monensin or Brefeldin A, completely abolished the α_v_β_3_ integrin-mediated SARS-CoV-2 cell entry. These data indicate that upon Spike binding to αvβ3, the complex is internalized by clathrin-mediated endocytosis.

## 4. Discussion

Viruses may utilize different receptors to broaden their range of target cells, thus resulting in variation of mechanism entry pathway into host cells [5].

Integrins belong to a family of heterodimeric transmembrane adhesion receptors comprising an α and a β subunits, which play a crucial role in cell adhesion, migration, invasion, growth, and survival [45]. As a minimum, 18 α and 8 β subunits were described to form 24 integrin pairs with different distributions and specificities [46]. Integrins are often used by many viruses as cellular receptors and the RGD (Arg-Gly-Asp) motif is the shortest sequence required for such interaction, being able to bind one or a combination of a variety of integrins [5].

Recently, we demonstrated that SARS-CoV-2 infects human primary HL-mECs by using integrins as an alternate receptor to ACE2 [11]. SARS-CoV-2/integrins interaction occurs through a conserved RGD motif (aa 403–405) that is present in the RBD of the Spike proteins of all SARS-CoV-2 lineages circulating to date, while it is absent in all the other coronavirus Spike proteins [9].

The SARS-CoV-2 virus underwent mutagenesis over time and some mutations are considered to be part of its adaptive evolution process. Among these, those acquired in the RBD of the Spike protein [47] and in the furin cleavage site [48,49] are believed to have increased Spike protein affinity to ACE2 and a more efficient viral replication. Indeed, the B.1.1.529 lineage, endowing multiple mutations in its Spike protein, is the most transmissible variant and thus favored by evolution. Likewise, the gaining of the polybasic site (PRRAR) at the junction of S1 and S2 cleavage site [50] may have allowed the cross-species transmissibility of the virus [38]. It is worth noting that the RGD motif may derive from a mutation in Spike at position 403 of the Bat coronavirus RaTG13 [51], one of the most closely related strain to SARS-CoV-2 [52]. Indeed, a point mutation in the second codon of “ACA” into “AGA” may have generated the “T403R” substitution that characterizes the SARS-CoV-2 integrin-binding motif. This evidence corroborates the hypothesis that the acquisition of the RGD motif may have conferred a crucial advantage in promoting zoonotic virus spillover from animals to humans and vice versa.

The potential clinical significance of the Spike protein holding the RGD motif in SARS-CoV-2 is notable and it is plausible that SARS-CoV-2 acquired integrin binding ability in order to widen its cellular targets when the ACE2 entry site is either poorly expressed or absent on the cell surface. At the same time, the RGD motif may represent an immune escape route always available when the ACE2 receptor is already engaged with antibodies elicited by natural infection or vaccination.

The concept of molecular mimicry of pathogens is not new. Indeed, it has been demonstrated that different viruses such as human immunodeficiency virus type-1 [53,54], Hepatitis C [55,56] and Influenza virus [57] possess proteins endowing sequence homologies with those of the host. This phenomenon allows viruses to evade host immune recognition by making functional epitopes of their proteins indistinguishable from the self-ones. The RGD region is highly conserved in human proteins for integrins’ interaction; thus, it is not surprising that SARS-CoV-2 utilizes the RGD motif to escape immune response and exert biological functions. Therefore, the evidence that antibodies evoked by the vaccination are not sufficient to inhibit integrins-mediated SARS-CoV-2 infection calls for the urgent need of intervention strategies aimed to neutralize the SARS-CoV-2 integrin-mediated infection of host cells in the vaccine era.

The common denominator of a main pathophysiological process in severe and long COVID-19 involves EC dysfunction, which is attested by the presence of abnormalities within the microvasculature, including prothrombotic phenotype, disseminated intravascular coagulation, and intussusceptive angiogenesis [20,21]. While ACE2 is highly expressed in human epithelial cells of the lung and small intestine [58], it is slightly or not expressed in most of the endothelial cells [11]. On the opposite, integrins have been found to be greatly expressed in a wide range of cells [59]. Our study identifies α_v_β_3_ integrin as the main receptor for SARS-CoV-2 entry into HL-mECs, thus establishing a mechanistic role for SARS-CoV-2-mediated integrin activation required for EC dysfunction. More specifically, here we show the presence of α_v_β_3_ integrin on the surface of HL-mECs and demonstrated that α_v_β_3_ integrin directly binds to the RBD of SARS-CoV-2 Spike protein by SPR analysis. After its binding to the HL-mECs surface, SARS-CoV-2 is internalized following a clathrin-dependent endocytic pathway and promotes EC dysfunction, mostly sustained by the secretion of a plethora of angiogenic molecules.

The inhibition of SARS-CoV-2/α_v_β_3_ interaction importantly prevented viral infection of HL-mECs and, more critically, was sufficient to counteract SARS-CoV-2-triggered angiogenesis. Since SARS-CoV-2 induces multiple clinical manifestations linked to integrins engagement [22], our findings corroborate the hypothesis that these receptors may likely contribute to the pathological states associated with the acute phase of COVID-19.

Although the virus has continuously evolved in order to enhance its fitness and adapt to the human host, it has maintained the RGD motif in all the variants emerged worldwide over time. This evidence suggests that this motif is a functional constraint, whose significance is not completely understood yet. In fact, beside its role in allowing SARS-CoV-2 entry into target cells by an alternate receptor, it probably represents a gain of functions aimed also to contribute to viral dissemination. Whatever the role of the acquisition of the RGD motif in the SARS-CoV-2 Spike protein is, it deserves to be deeper investigated and, unquestionably, needs to be inhibited.

Integrins represent attractive therapeutic targets, their inhibitors of function having been successfully tested as drugs to treat several pathological conditions [60]. Moreover, several antagonists of α_v_β_3_ integrin have been tested as antiangiogenic molecules [61]. Our data highlight the use of integrin inhibitors to disrupt virus-cell interaction and mitigate some of the worst clinical features of the severe form of COVID-19 related to integrin engagement by SARS-CoV-2. Although integrins seem to represent a promising approach to limit viral pathogenesis, additional studies are necessary to decipher these data into clinical practice.

## Data Availability

Genomic data reported in this study are available at Global Initiative on Sharing All Influenza Data (GISAID). Accession numbers: EPI_ISL_1379197; EPI_ISL_ 7284686; EPI_ISL_1379435; EPI_ISL_1379437; EPI_ISL_1379439; EPI_ISL_1379442; EPI_ISL_3098727.
